# Single-cell transcriptomics reveals heterogeneity in esophageal squamous epithelial cells and constructs models for predicting patient prognosis and immunotherapy

**DOI:** 10.3389/fimmu.2023.1322147

**Published:** 2023-11-30

**Authors:** Chenglin Li, Wei Song, Jialing Zhang, Yonggang Luo

**Affiliations:** ^1^ Department of Cardiothoracic Surgery, The Affiliated Huaian No.1 People’s Hospital of Nanjing Medical University, Huaian, Jiangsu, China; ^2^ Department of Gastroenterology, The Affiliated Huaian No.1 People’s Hospital of Nanjing Medical University, Huaian, Jiangsu, China

**Keywords:** ESCC, tumor microenvironment, immunotherapy, prognosis, signature

## Abstract

**Background:**

Esophageal squamous cell carcinoma (ESCC), characterized by its high invasiveness and malignant potential, has long been a formidable challenge in terms of treatment.

**Methods:**

A variety of advanced analytical techniques are employed, including single-cell RNA sequencing (scRNA-seq), cell trajectory inference, transcription factor regulatory network analysis, GSVA enrichment analysis, mutation profile construction, and the inference of potential immunotherapeutic drugs. The purpose is to conduct a more comprehensive exploration of the heterogeneity among malignant squamous epithelial cell subgroups within the ESCC microenvironment and establish a model for predicting the prognosis and immunotherapy outcomes of ESCC patients.

**Results:**

An analysis was conducted through scRNA-seq, and three Cluster of malignant epithelial cells were identified using the infer CNV method. Cluster 0 was found to exhibit high invasiveness, whereas Cluster 1 displayed prominent characteristics associated with epithelial-mesenchymal transition. Confirmation of these findings was provided through cell trajectory analysis, which positioned Cluster 0 at the initiation stage of development and Cluster 1 at the final developmental stage. The abundance of Cluster 0-2 groups in TCGA-LUAD samples was assessed using ssGSEA and subsequently categorized into high and low-expression groups. Notably, it was observed that Cluster 0-1 had a significant impact on survival (p<0.05). Furthermore, GSVA enrichment analysis demonstrated heightened activity in hallmark pathways for Cluster 0, whereas Cluster 1 exhibited notable enrichment in pathways related to cell proliferation. It is noteworthy that a prognostic model was established utilizing feature genes from Cluster 0-1, employing the Lasso and stepwise regression methods. The results revealed that in TCGA and GSE53624 cohorts, the low-risk group demonstrated significantly higher overall survival and increased levels of immune infiltration. An examination of four external immunotherapy cohorts unveiled that the low-risk group exhibited improved immunotherapeutic efficacy. Additionally, more meaningful treatment options were identified for the low-risk group.

**Conclusion:**

The findings revealed distinct interactions between malignant epithelial cells of ESCC and subgroups within the tumor microenvironment. Two cell clusters, strongly linked to survival, were pinpointed, and a signature was formulated. This signature is expected to play a crucial role in identifying and advancing precision medicine approaches for the treatment of ESCC.

## 1 Introduction

Esophageal cancer(EC), a prevalent malignant neoplasm affecting populations worldwide, exhibits alarmingly high incidence and mortality rates. The year 2020 alone witnessed a staggering 604,000 newly diagnosed cases of EC, tragically resulting in 544,000 fatalities (1). This formidable disease encompasses two predominant pathological classifications: esophageal adenocarcinoma and esophageal squamous carcinoma (ESCC), with ESCC representing the predominant subtype among new patients each year (2). Despite notable advancements in scientific and technological domains, the therapeutic armamentarium for EC has expanded considerably. However, the overall prognosis remains disheartening, as evidenced by a discouraging 5-year survival rate ranging between a mere 10% and 30% (3, 4). Furthermore, extensive research has unveiled substantial variations in surgical and pharmacological responses among patients sharing the same clinical stage, thus highlighting pronounced prognostic heterogeneity. This phenomenon primarily stems from the current reliance on TNM staging, widely employed in clinical practice, which regrettably neglects the cellular and even molecular disparities exhibited by these patients (5).

Esophageal Cancer Epithelial Cells Heterogeneity (HECEC) encompasses the intricate diversity and variability observed among epithelial cells within the tissue of EC. This heterogeneity manifests at the molecular level, characterized by disparities in gene expression and protein profiles. Distinct subpopulations of epithelial cells exhibit specific gene expression patterns, and scrutinizing these differences in gene and protein expression unveils the molecular attributes and potential driving mechanisms unique to each subpopulation (4, 6). HECEC exerts a profound impact on the development, metastasis, treatment, and prognosis of esophageal cancer. Varied subpopulations of cells may demonstrate disparate sensitivities and resistances to therapeutic agents, highlighting the significance of tailoring individualized treatment strategies. Consequently, conducting an in-depth exploration of epithelial cell heterogeneity in EC becomes paramount, as it unveils the molecular features and functional properties inherent to distinct subpopulations. This research serves as a crucial foundation for providing personalized treatments and improving the prognosis of individuals afflicted with EC (2, 7).

The advent of single-cell RNA sequencing (scRNA-seq) has revolutionized the field by offering a formidable tool for delving into the intricacies of tumor heterogeneity. Traditional bulk RNA-seq technology falls short in capturing the nuanced heterogeneity at the transcriptional level, limiting our understanding of intratumor heterogeneity and the intricate tumor microenvironment (TME). In contrast, the emerging technique of scRNA-seq boasts remarkable advantages such as high throughput and efficiency. Leveraging these benefits, scRNA-seq enables the identification of molecular features within tumors, decoding the intricate landscape of intratumor heterogeneity, and unearthing novel therapeutic targets and clinical biomarkers (8, 9).

Our study utilized scRNA-seq and bulk RNA-seq datasets to dissect the heterogeneity of ESCC epithelial cells. By categorizing different cancer epithelial clusters, we investigated their crucial roles within the TME. Ultimately, we constructed a signature using key cancer epithelial subgroups that can predict the prognosis and response to immunotherapy for ESCC patients. This provides valuable insights for the clinical stratification and treatment of ESCC patients.

## 2 Methods

### 2.1 Dataset source

The acquisition of bulk RNA-seq data, mutation data, and clinical characteristics related to ESCC patients diagnosed was facilitated through the utilization of The Cancer Genome Atlas (TCGA) database (https://portal.gdc.cancer.gov). Additionally, a scRNA-seq dataset (GSE188900) (10), comprising samples from six ESCC patients, including seven surgically resected tumor tissue specimens and one healthy tissue specimen, was obtained from the Gene Expression Omnibus (GEO) database (http://www.ncbi.nlm.nih.gov/geo). Furthermore, four datasets related to immunotherapy were aggregated from the GEO database, encompassing comprehensive transcriptomic data and the responses of patients to immunotherapy, as described below:

GSE91061: Nivolumab therapy was administered to 65 patients with advanced-stage melanoma (11).

GSE100797: This dataset consisted of 27 stage IV melanoma patients who participated in ACT clinical phase I/II trials (12).

GSE126044: Sixteen patients with non-small-cell lung cancer underwent PD-1 therapy (12).

GSE35640: It included 65 melanoma patients who were enrolled in a phase II trial involving recombinant MAGE-A3 antigen combined with an immunological adjuvant (13).

These data resources have been effectively utilized to provide robust support for our research, enabling a comprehensive understanding of the molecular characteristics of ESCC patients and their responses to immunotherapy. To ensure data uniformity and comparability, the expression data was transformed into the Transcripts Per Million (TPM) format, and potential batch effects were mitigated using the “combat” function within the “sva” R package (14). Furthermore, all data from the TCGA database, including bulk sequencing data, mutation data, and clinical details of ESCC patients, were logarithmically transformed to achieve a standardized data format before the initiation of the analysis.

### 2.2 The detailed steps of the single-cell analysis process

In single-cell RNA sequencing analysis, we utilized the Seurat R package (15, 16) (version 4.2.0) to transform the raw data into a Seurat object. During the data preprocessing, we implemented stringent quality control measures. Specifically, we excluded cells that expressed fewer than 300 genes or more than 5,000 genes, as well as cells in which the UMIs originating from the mitochondrial genome accounted for more than 10% of the total UMIs. To reduce data dimensionality, we performed Principal Component Analysis (PCA) on the variably expressed genes, selecting the top 20 principal components. Subsequently, we conducted clustering using the “FindCluster” function with a resolution parameter set to 0.5, and visualized the results using UMAP. To identify marker genes for distinct cell clusters, we employed Seurat’s “FindAllMarkers” function, comparing cells within a specific cluster to all other cells. Through the use of canonical marker genes, we annotated the cell clusters in the resulting two-dimensional representation with known biological cell types. It is worth noting that, in the analysis, we chose not to correct for cell cycle effects, as only a limited number of cells exhibited positive expression of cell proliferation markers.

### 2.3 Infer the malignant squamous epithelial cells

The InferCNV approach (17) was employed to validate copy number variations (CNVs) and discern between malignant cells and normal epithelial cells. To construct trajectories, high CNV score epithelial cells were extracted from squamous epithelial cells and designated as cancerous epithelial cells. Subsequently, the Monocle2 algorithm was employed (18), using a gene-cell matrix extracted from a Seurat subset with UMI counts scaled, as input. Default parameters were applied to infer cellular trajectories.

### 2.4 GSVA enrichment analysis

A gene set enrichment analysis was conducted using 50 hallmark pathways from the Molecular Signatures Database (MSigDB). To assign pathway activity estimates to each cell type, Gene Set Variation Analysis (GSVA) was performed on each cell, followed by calculating the average gene expression levels for each cell subtype, utilizing the standard settings in the GSVA package (19). Differences between activity scores were used to quantify differential pathway activity among distinct cell subtypes.

### 2.5 Cell-cell communication and inference of transcription factors

We integrated gene expression data using CellChat (20) to assess differences in hypothesized cell-cell communication modules. Following the standard CellChat pipeline, we employed the default CellChatDB as the ligand-receptor database. Cell type-specific interactions were inferred by identifying overexpressed ligands or receptors within a cell group, followed by the identification of enhanced ligand-receptor interactions when ligands or receptors were overexpressed. Additionally, the R package Scenic was utilized to infer the activity of gene regulatory networks.

### 2.6 Gene regulatory networks

The R software package Scenic is employed to deduce the functioning of gene regulatory networks. The default settings are utilized to assess the activity of individual regulators in single cells, drawing upon the cisTarget databases: hg38_refseq-r80_500bp_up_and_100bp_down_tss.mc9nr.feather and hg38_refseq-r80_10kb_up_and_down_tss.mc9nr.feather.

### 2.7 Building the high-performance epithelial-associated signature (EAS) of ESCC

Univariate Cox regression analysis was utilized to evaluate the influence of these genes on the survival status of ESCC. To minimize the risk of overlooking significant factors, we set the cutoff P-value to 0.05. Following this, we applied the LASSO Cox regression method (21) to reduce the number of candidate genes, ultimately creating the most optimal survival signature. The model’s predictive performance was evaluated using receiver operating characteristic (ROC) curves, with an area under the curve (AUC) value exceeding 0.65 indicating exceptional performance.

### 2.8 Mutation landscape

A comprehensive analysis of somatic mutation frequency and distribution across a range of genes was conducted utilizing the “maftools” R package (22). Concurrently, TCGA-ESCC patients were subjected to a stratification process, resulting in their classification into four distinct groups based on their median risk score and median tumor mutational burden (TMB). Subsequently, a comparative analysis was executed to scrutinize disparities in survival among these groups, contingent upon their respective median risk scores and TMB values.

### 2.9 Differences in the TME and drug inference

The efficacy of tumor immunotherapy is influenced by the complex TME (23, 24). Six different immune infiltration algorithms were employed to rigorously assess the composition of immune cells within distinct risk groups. Subsequently, to convey the intricate variances in immune cell infiltration across these risk groups, heatmaps were utilized as effective visual tools, thus elucidating subtle differences among immune cell populations. Additionally, the specialized functionalities of the “estimate” R package (25) were meticulously utilized to quantify immune scores, stromal scores, and ESTIMATE scores for patients diagnosed with ESCC. This strategic deployment enhanced a comprehensive evaluation of the TME and its potential implications. In the pursuit of identifying potentially effective chemotherapeutic agents among different risk groups, the predictive capabilities provided by the “oncoPredict” R package (26) were extensively utilized. Through the application of this tool, a profound prediction of suitable therapeutic interventions was enabled, contributing to a more informed treatment strategy.

### 2.10 SubMap validation

The significance of shared characteristics between two groups is evaluated using the unsupervised method, SubMap, with a significance threshold denoted by an adjusted p-value below 0.05, indicating substantial similarity. Subtype consistency between the validation and discovery cohorts was assessed utilizing the SubMap approach, and the results were subsequently presented through the ComplexHeatmap package.

### 2.11 Collection of clinical samples and cell lines and ethics

Ethical approval was obtained from the Medical Ethics Committee at The Affiliated Huaian No.1 People's Hospital of Nanjing Medical University to collect tissue specimens. These specimens, which included both tumor (T) and precancerous (N) tissues from patients with ESCC who had undergone tumor resection, were carefully stored at -80°C. TE1 and KYSE30, human esophageal squamous cell carcinoma (ESCC) cell lines, were obtained from the Cell Resource Center at the Shanghai Life Sciences Institute. The extraction of total RNA from ESCC tissues was performed using the TRIzol reagent from Thermo Fisher Scientific, headquartered in Waltham, MA, USA. Subsequently, cDNA synthesis followed the manufacturer’s instructions, utilizing the RevertAid™ First Strand cDNA Synthesis Kit, also provided by Thermo Fisher Scientific. The qRT-PCR analysis was conducted using the StepOne Real-Time PCR system, an instrument also manufactured by Thermo Fisher Scientific. For amplification, the SYBR Green PCR kit from Takara Bio in Otsu, Japan, was utilized. The quantification of relative gene expression levels was achieved through the 2^-△△CT^ method.

### 2.12 Colony formation

For colony formation analysis, 1000 cells were transfected and cultured in 6-well plates for approximately 14 days. After this period, cell clones were visually examined without magnification. The cells were then washed, fixed with 4% paraformaldehyde (PFA) for 15 minutes, stained with crystal violet from Solarbio, China, for 20 minutes, and air-dried at room temperature. The cell count per well was then determined.

### 2.13 Statistical analysis

R 4.2.0 software was employed for all data processing, statistical analysis, and visualization. Subtype-specific overall survival (OS) was estimated and compared using the Kaplan-Meier method and log-rank test. Differences in continuous variables between the two groups were assessed via the Wilcoxon test or t-test. For categorical variables, the analysis was performed using the chi-squared test or Fisher’s exact test. The false discovery rate (FDR) method was utilized to correct p-values. Correlations between variables were assessed through Pearson correlation analysis. All p-values were calculated with a two-tailed approach, with statistical significance defined as p < 0.05.

## 3 Results

**Figure 1:**
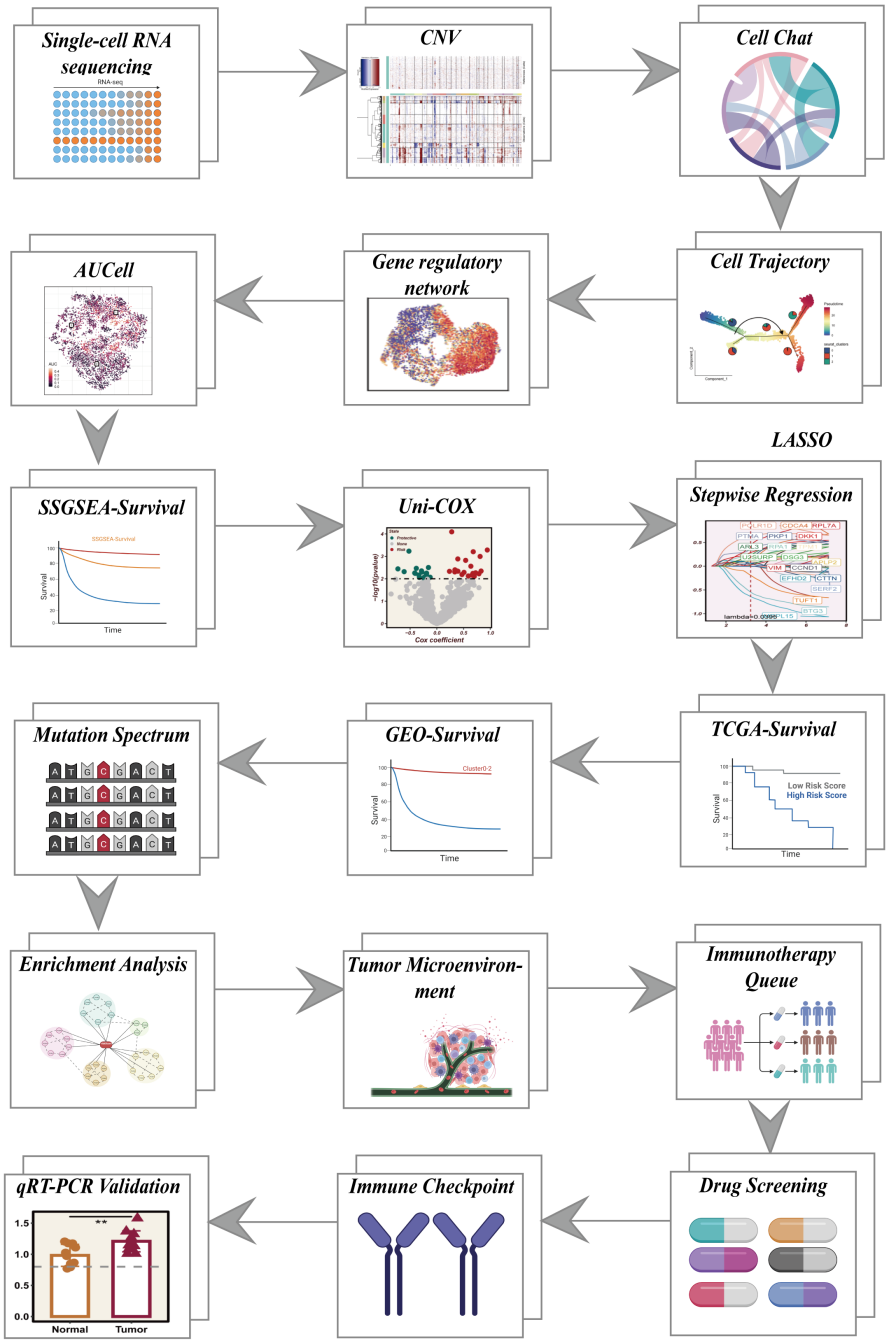
Overall flowchart of all analyses.

**Figure 1** shows a flow chart outlining the study.

### 3.1 The scRNA profiling of LUAD

**Figure 2:**
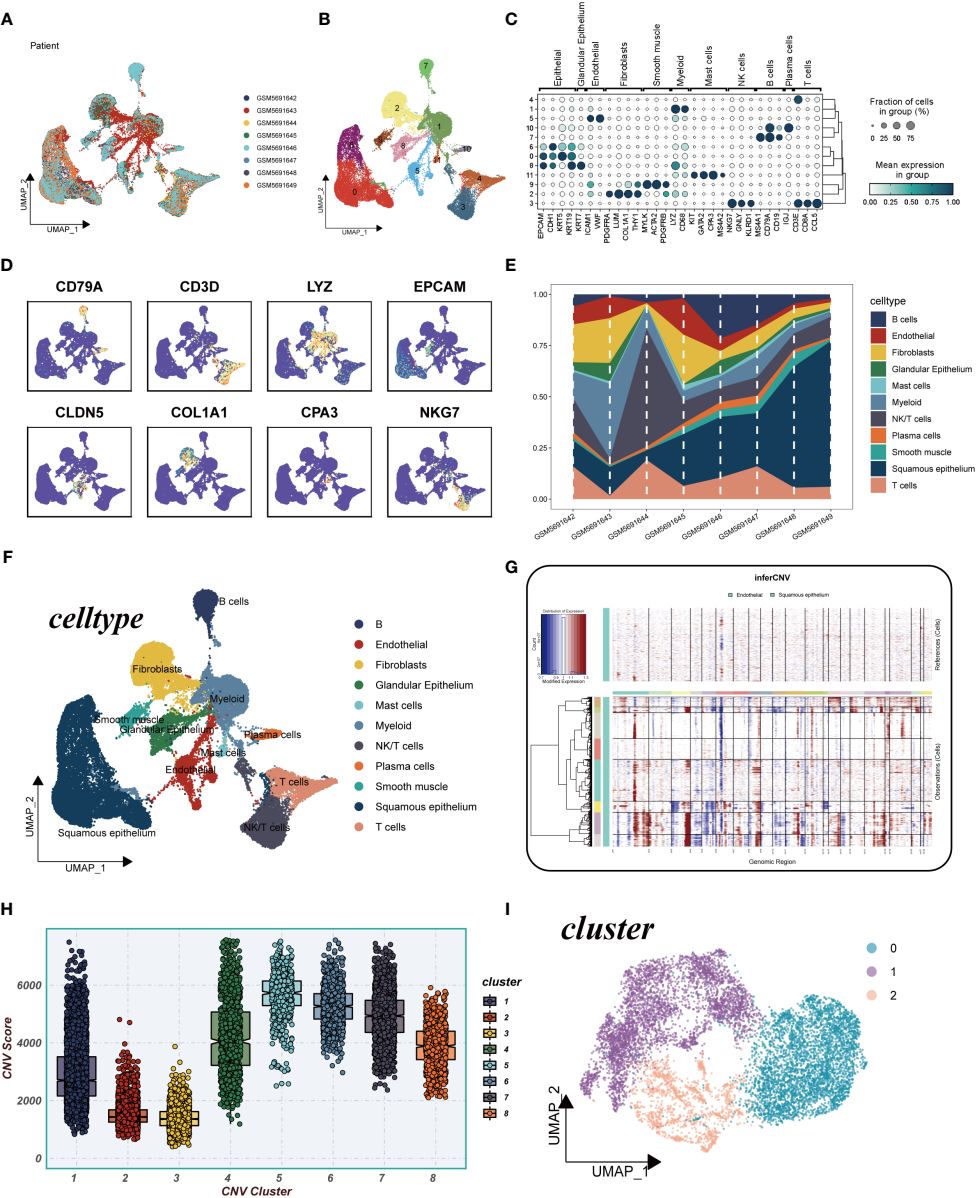
Explanation of cellular subpopulations. **(A)** Excluding batch effects between samples. **(B)** UMAP plot for descending cluster sorting. **(C)** Bubble plot of mean expression of marker genes for each cell type. **(D)** UMAP plot reveals marker gene expression levels across diverse cell types. **(E)** Proportions of 11 cell types originated from different tissues. **(F)** Cellular annotations unveil 11 distinct cell phenotypes. **(G)** Analysis of copy number loss or amplification of each chromosome in endothelial and squamous epithelial cells by InferCNV algorithm. **(H)** Comparison of CNV score for 8 clusters. **(I)** UMAP plot for all squamous epithelial cells clustered into four clusters.

This study encompassed a total of 8 samples, each exhibiting a relatively stable cell distribution, suggesting minimal susceptibility to batch effects. Consequently, these samples served as a robust foundation for subsequent analyses (**Figure 2A**). Leveraging the UMAP algorithm, all cells were meticulously categorized into 12 clusters, providing a detailed classification (**Figure 2B**). The comprehensive bubble plots depicted in **Figure 2C** illustrated the expression patterns of characterization marker genes associated with each of the 11 cell clusters. Cell type identification relied on the marker genes showcased in **Figure 2D**. To assess the distribution proportions of these 11 cell types across the 8 samples, **Figure 2E** presented the corresponding proportions. Intriguingly, **Figure 2F** unveiled the existence of diverse cell types, including squamous epithelial cells, T cells, and smooth muscle cells, among others. Moreover, through the application of inferCNV, **Figure 2G** elucidated the identification of individual chromosomes, with squamous epithelial cells demonstrating higher CNVs compared to endothelial cells in most instances. Notably, significant copy number deletions were observed on chromosome 6 in almost all tumor cells. To explore the distributional disparities in CNV scores among the eight clusters, **Figure 2H** highlighted the selection of cluster 1, cluster 4, and cluster 5-8, characterized by elevated copy number variations. Moreover, squamous epithelial cells within these clusters underwent UMAP downscaling, enabling their classification into three distinct subclusters: Cluster 0, Cluster 1, and Cluster 2 (**Figure 2I**).

### 3.2 Trajectory analysis and cellular communication

**Figure 3:**
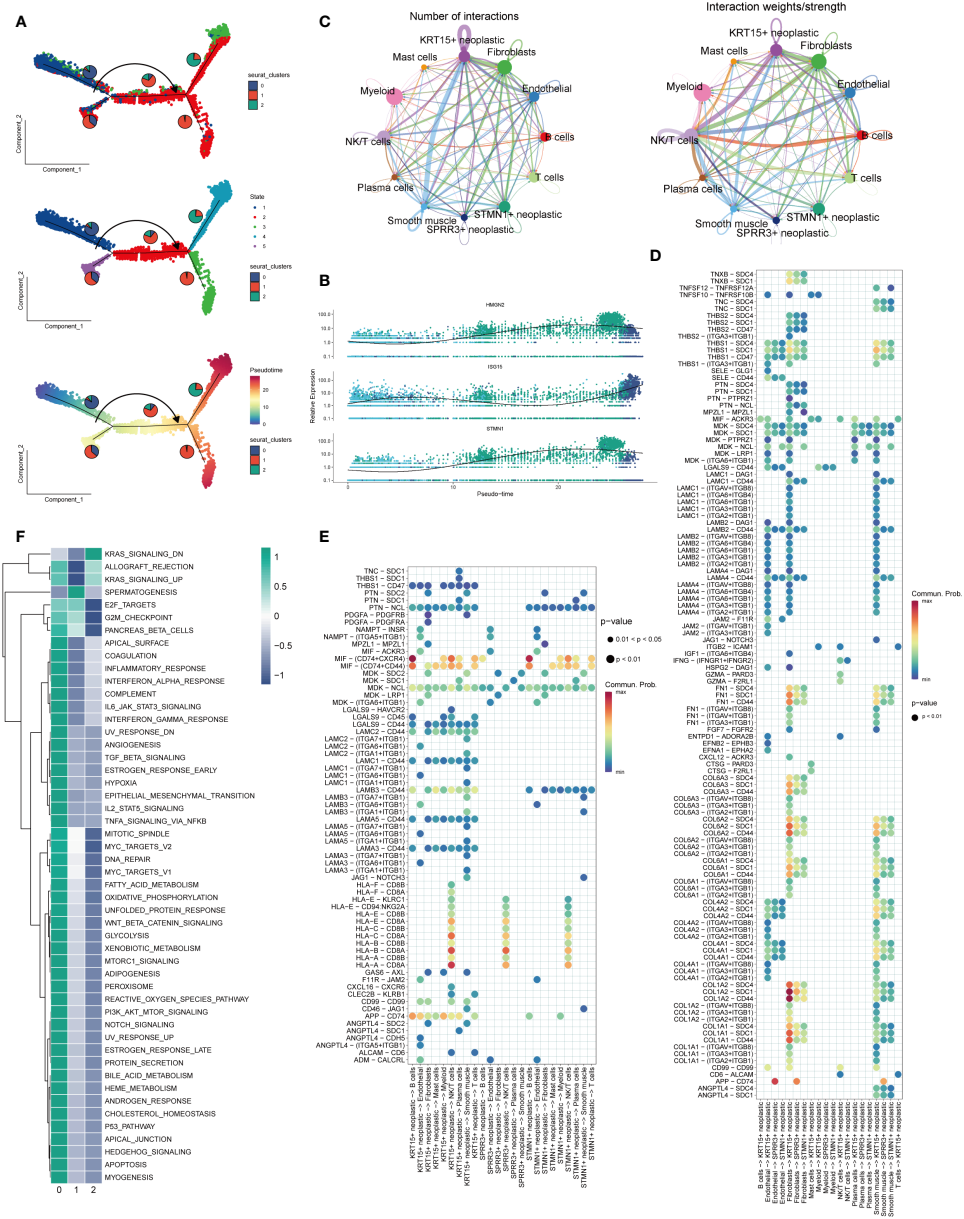
Trajectory analysis and cellular interactions analysis. **(A)** All squamous epithelial cells’ differentiation trajectories, pseudotime distribution, and cell clusters on pseudotime and the proportion of each clusters. **(B)** Relative expression of HMGN2,ISG15 and STMN1 in pseudo-time. **(C)** Number and strength of cellular communications between KRT15+ neoplastic, SPRR3+ neoplastic, STMN1+neoplastic and other type cells. **(D)** KRT15+ neoplastic, SPRR3+ neoplastic and STMN1+neoplastic acting on different cells ligand-receptor bubble diagram. **(E)** Ligand-receptor bubble diagram of different types of cells acting on KRT15+ neoplastic, SPRR3+ neoplastic and STMN1+neoplastic. **(F)** Enrichment analysis of the three clusters.

In **Figure 3A**, cellular transcriptional heterogeneity in malignant squamous epithelial cells was assessed via trajectory analysis using the “monocle2” R package. Over the pseudotime progression, there was a gradual reduction in the prevalence of the cluster0 subtype, concomitant with a progressive augmentation in the proportions of cluster1 and cluster2 subtypes. **Figure 3B** showcases the relative expression of the three most significantly altered genes, namely HMGN2, ISG15, and STMN1, represented in pseudo time. This representation provides insights into the temporal dynamics of gene expression changes. In **Figure 3C**, the illustration demonstrates the quantity and intensity of cellular communication between KRT15+ neoplastic cells (Cluster0), STMN1+ neoplastic cells (Cluster1), SPRR3+ neoplastic cells (Cluster2), and other cell types within ESCC tissues. This visualization sheds light on the intricate network of intercellular interactions. Furthermore, in **Figures 3D, E**, we delved into the ligand-receptor interactions existing between different cell types and the three labeled tumor cells within ESCC tissues. Notably, we discovered that KRT15+ neoplastic cells engaged with other cell types through the APP-CD74, MIF-(CD74 + CXCR4), and MIF-(CD74 + CD44) receptor-ligand pairs. Similarly, STMN1+ neoplastic cells also established contacts with other cells via the MIF-(CD74 + CXCR4) and MIF-(CD74 + CD44) receptor-ligand pairs. Additionally, fibroblast cells and smooth muscle cells exhibited the ability to communicate with KRT15+ neoplastic cells through several ligand-receptor pairs. Moreover, in **Figure 3F**, we conducted an analysis to assess the enrichment of the three identified cell clusters. Cluster 0 displayed enrichment across nearly all channels, indicating its prominence across multiple biological processes. Conversely, Cluster 1 showed enrichment primarily in spermatogenesis-related channels, while Cluster 2 exhibited enrichment specifically in the down-regulated KRAS signaling pathway.

### 3.3 Regulon prediction

**Figure 4:**
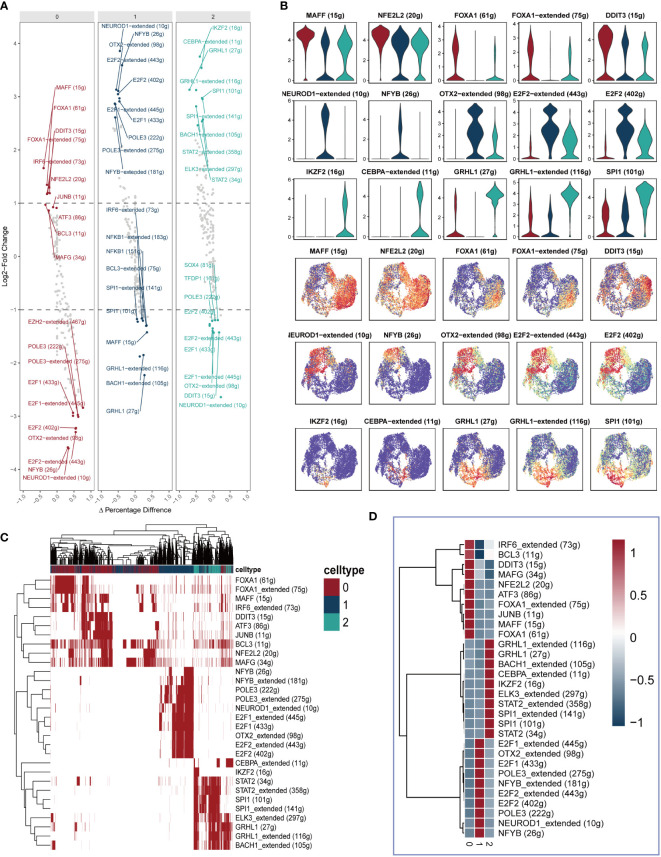
Identification of differently expressed gene regulatory elements. **(A)** The first 10 highly expressed genes and the first 10 lowly expressed genes in each cluster. **(B)** The expression of five genes in each cluster were showed in Violin plot and UMAP plot. **(C, D)** Heatmap presenting the distribution of gene regulatory elements in different clusters.

**Figure 4A** presents the top 10 gene regulatory regulars that exhibit high expression levels as well as the top 10 gene regulatory regulars with low expression levels for each cell cluster. This analysis offers insights into the differential gene expression patterns within each cluster. Subsequently, **Figure 4B** showcases the expression of five selected gene regulatory regulars within each cluster, with their locations indicated on the UMAP plots. Furthermore, **Supplementary Figure 1** provides a comprehensive view of the gene expression profiles within each cluster, displaying the expression patterns of specific genes. To further elucidate the differential gene expression patterns, **Figures 4C, D** present heatmaps illustrating the differential expression of the top 10 gene regulatory elements across all cells within each of the three cell clusters. These heatmaps provide a visual representation of the variations in gene expression, highlighting the distinctive expression patterns specific to each cluster.

### 3.4 Aggressive and EMT score

**Figure 5:**
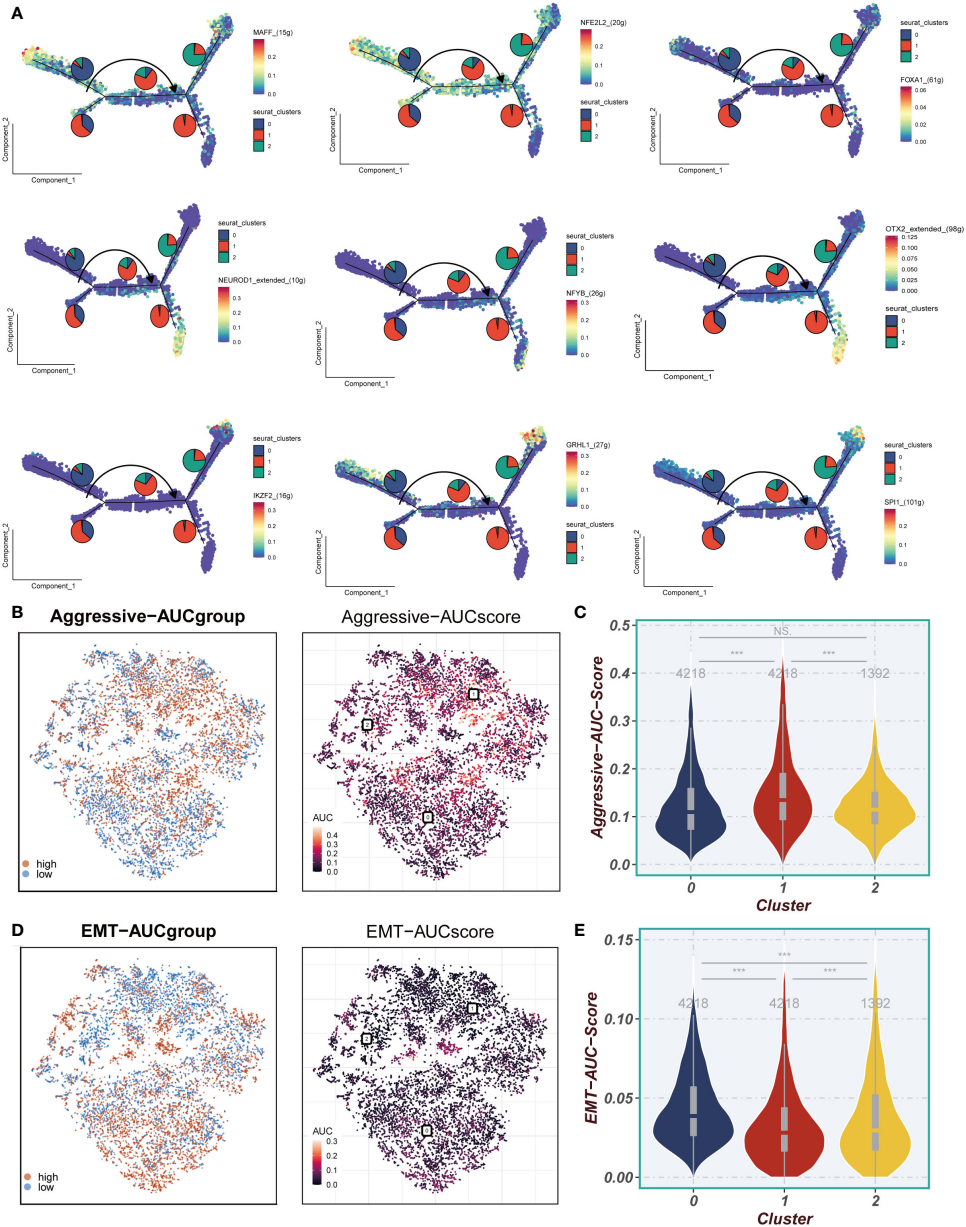
Invasion and EMT Features. **(A)** The cell trajectory analysis of different regulons. **(B, C)** Aggressive levels of three clusters were showed in UMAP plot and Violin plot. **(D, E)** EMT levels of three clusters were showed in UMAP plot and Violin plot. ***P < 0.001; ns, P < 0.05.

In **Figure 5A**, the transcription factors displaying the highest specificity for Cluster 0-2 epithelial cell subgroups were integrated into the pseudotime inference analysis. Notably, MAFF, NFE2L2, and FOXA1 were observed to be upregulated in Cluster 0, while NEUROD1, NFYB, and OTX2 exhibited upregulation in Cluster 1, and IKZF2, GRHL1, and SPI1 showed elevated expression in Cluster 2. Moving to **Figures 5B, C**, our analysis demonstrated that the Cluster 1 subpopulation displayed a notably higher Aggressive score compared to other cell subpopulations. This observation suggests an enhanced invasive ability of ESCC cells within the Cluster 1 subpopulation. Furthermore, as illustrated in **Figures 5D, E**, a substantial difference in the Epithelial-Mesenchymal Transition (EMT) score between Cluster 0 and Cluster 1/Cluster 2 was identified. Specifically, the EMT score of Cluster 0 was significantly higher than that of Cluster 1 and Cluster 2. This disparity implies that the esophageal cancer epithelial cells within the Cluster 0 subpopulation exhibit a more pronounced migratory ability, potentially associated with an increased propensity for metastasis.

### 3.5 Model developing and evaluating

**Figure 6:**
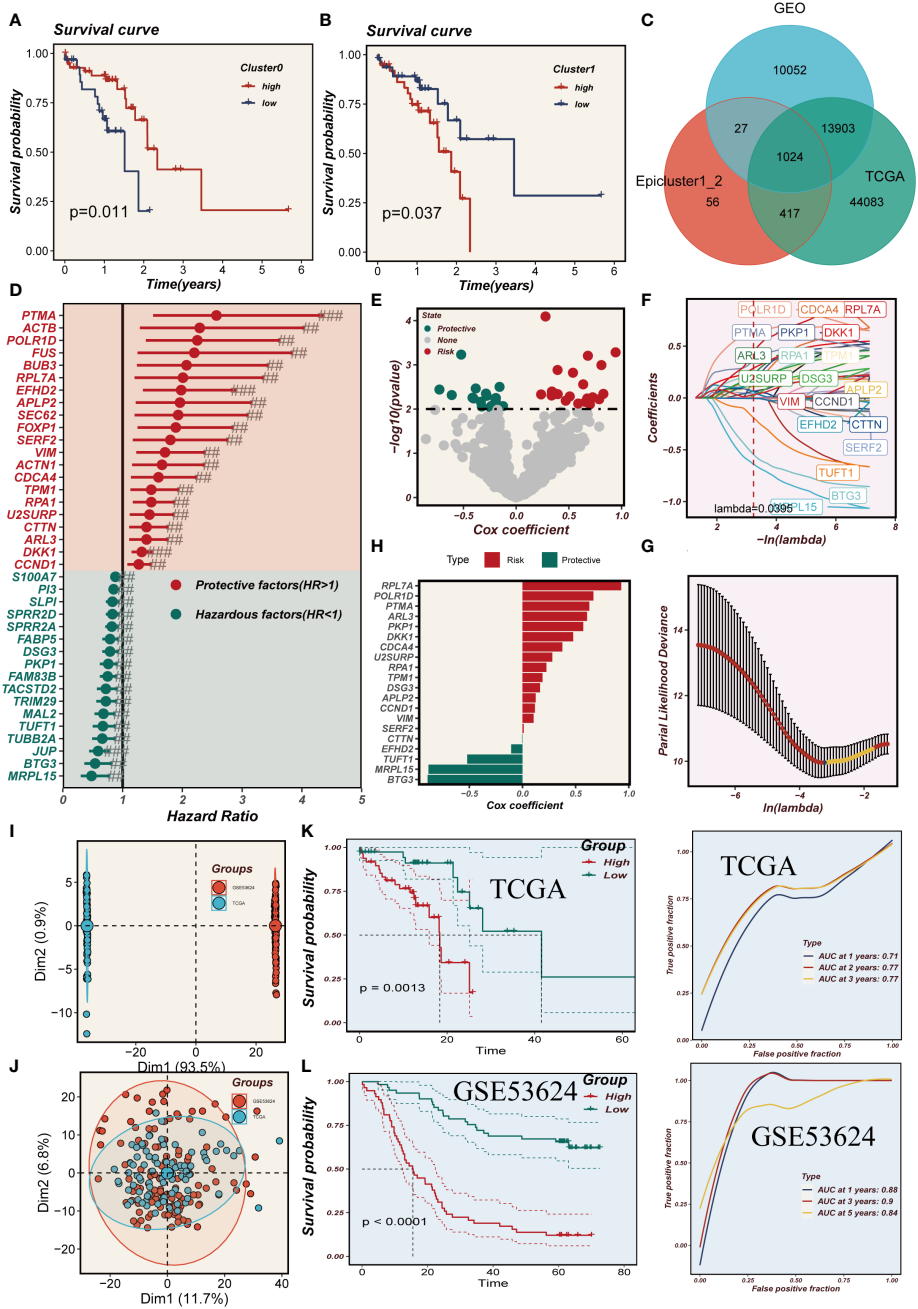
Model developing and evaluating. **(A, B)** The effect of cluster0 and 1 abundance on survival. **(C)** Venn diagram showing intersection genes of Epicluster0_1 with GEO and TCGA cohorts. **(D)** Forest plot shows the results of univariate COX analysis. **(E)** Volcano plot showing up- and down-regulated differential genes in the cohort. **(F, G)** LASSO regression screening for important prognosis-related genes. **(H)** Distribution of coefficient values of model genes. **(I, J)** Discernible batch effect detected in TCGA and GSE53624 cohorts, ensuring harmonized data integration by mitigating batch effects. **(K, L)** Differences in survival between the high and low risk groups in the TCGA and GSE53624 cohorts are presented separately, along with their ROC curves.

Based on the marker genes associated with Cluster 0-1, we utilized the ssgsea algorithm to assess their abundance in TCGA samples. We compared the survival outcomes between high and low abundances and found that a high abundance of Cluster 0 is indicative of better survival, whereas a high abundance of Cluster 1 is associated with poorer survival (**Figures 6A, B**). In **Figure 6C**, by intersecting cluster-identified genes in TCGA, GEO and cluster0-1, we identified a total of 1024 mark genes associated with the grouping of epithelial cell subpopulations in ESCC. A model was constructed using the training set of TCGA, and 38 prognostic genes were identified by univariate COX analysis (P<0.01). The results were presented using a forest plot to visualize the 21 protective factors and 17 risk factors (**Figure 6D**). Subsequently, the EAS was developed using LASSO and multifactorial Cox regression analyses, incorporating a total of 20 genes (**Figures 6E-H**). In **Figure 6I**, we observed a significant batch effect in the TCGA and GSE53624 independent cohorts, which were de-batched to obtain eligible cohorts for subsequent analysis (**Figure 6J**). Survival analysis showed that the prognosis of the high-EAS group in TCGA was significantly worse than that of the low-EAS group, a finding that was well validated in the GSE53624 cohort. Meanwhile, ROC curves were evaluated for the model, and it was found that the model had good predictive performance for the prognosis of esophageal cancer patients (**Figures 6K, L**).

### 3.6 Immune infiltration analysis

**Figure 7:**
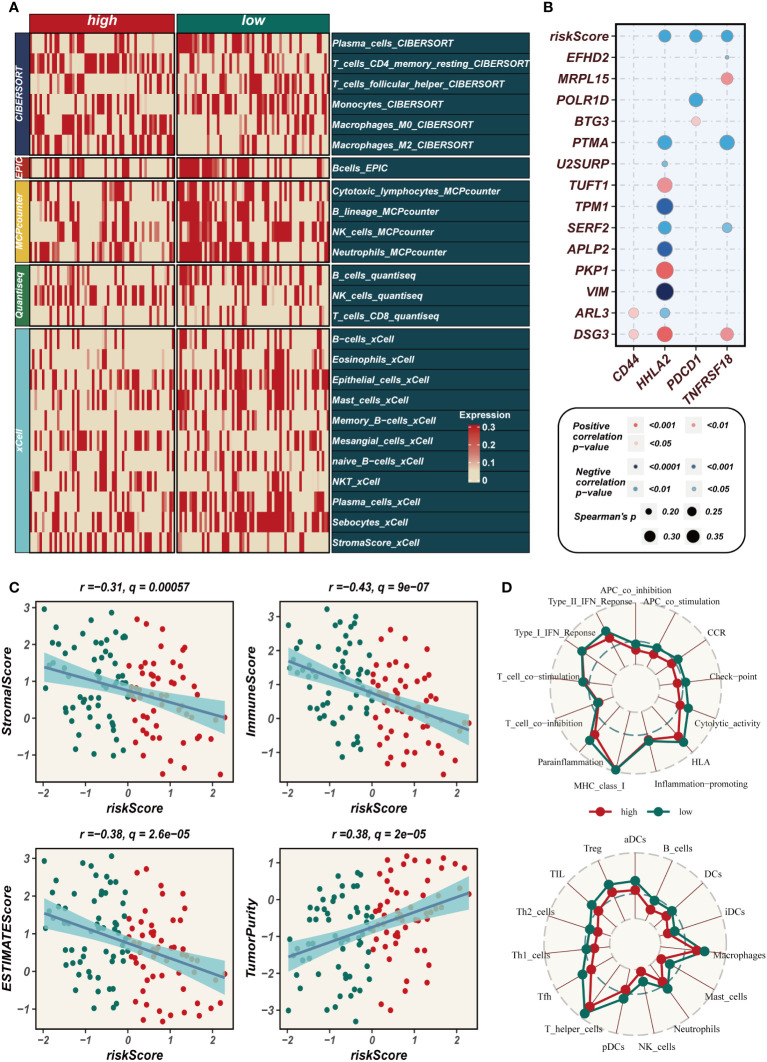
Assessment of immune infiltration. **(A)** Heat map showing the differences in immune cell infiltration between two risk groups assessed using five algorithms. **(B)** Bubble plots demonstrating the correlation between riskScore and part of model genes and immune checkpoint expression. **(C)** Scatter plot elucidates the correlation between risk score and stromal score, immune score, ESTIMATE score, and tumor purity, revealing intricate interconnections within the tumor microenvironment. **(D)** SsGSEA enrichment analysis shows high and low risk groups in terms of immune cell infiltration and enrichment of immune-related pathways.

The heat map depicted in **Figure 7A** employed five distinct methodologies to evaluate the extent of immune cell infiltration in both high- and low-EAS group. The findings indicated that immune cell infiltration was more pronounced in the low-EAS group. **Figure 7B** conducted an assessment of the association between CD44, HHLA2, PDCD1, and TNFRSF18 with the risk score, as well as with several modeled genes. The results demonstrated a significant correlation between HHLA2 and the risk score, as well as with some of the modeled genes. Furthermore, the risk score exhibited a negative correlation with HHLA2, PDCD1, and TNFRSF18. The “ESTIMATE” R software package was employed to gauge the level of immune infiltration, and subsequent correlation analysis revealed a noteworthy negative correlation between the risk score and the immune score. Conversely, a positive correlation was observed between the risk score and tumor purity (**Figure 7C**). To assess discrepancies in immune cell infiltration and immune-related pathways between the high- and low-EAS group, the ssGSEA method was utilized. The outcomes unveiled that the low-EAS group exhibited heightened levels of immune cell infiltration, encompassing NK cells, aDCs, and macrophages. Additionally, the low-EAS group manifested greater activity in numerous immune-related pathways, such as CCR, cytolytic activity, type I IFN response, among others (**Figure 7D**).

### 3.7 TMB and immunotherapy cohort

**Figure 8:**
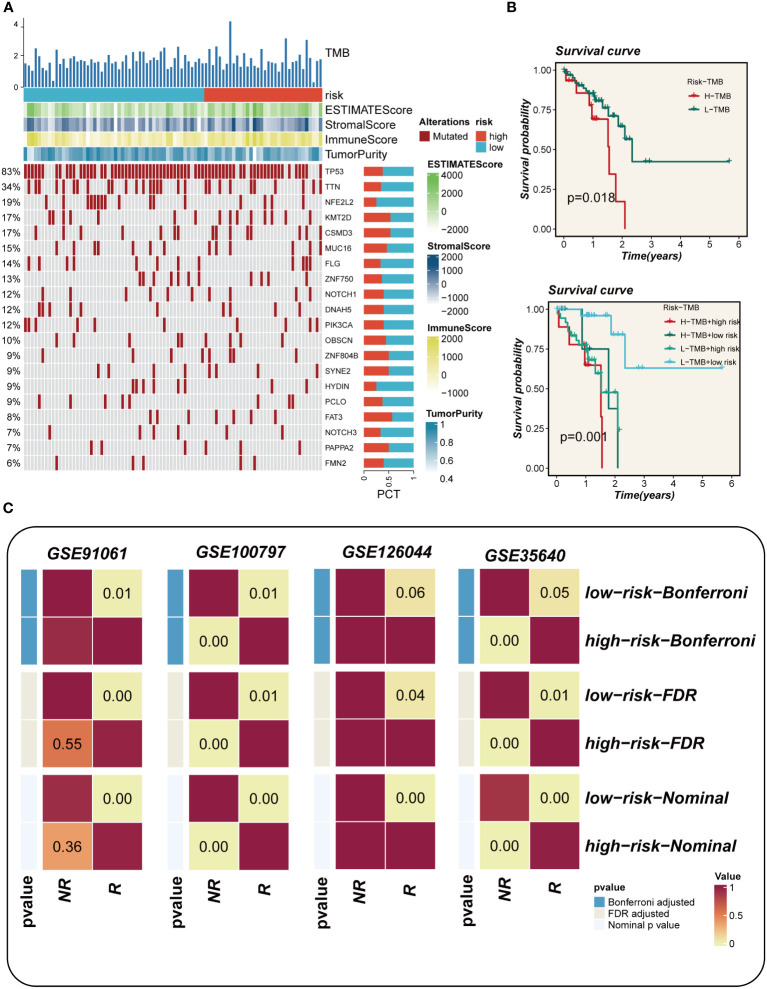
Mutation landscape analysis. **(A)** Waterfall plots depicting differences in frequently mutated genes for esophageal cancer in high and low risk groups. The left panel shows mutation rates, with genes sorted by mutation frequency. **(B)** Survival curves showing the difference between survival among different subgroups. **(C)** Subgraph analysis of the GEO dataset to assess the association between EAS and response to immunotherapy.

The waterfall plot presented in **Figure 8A**, which compared representative gene variants in the high- and low-EAS group, unveiled that TP53, TTN, MUC16, CSMD3, and RYR2 were the five genes exhibiting the highest frequency of variants. Notably, there was no discernible visual distinction in tumor mutational burden (TMB) between the two groups, as observed in the heat map. However, when patients were stratified based on TMB levels, it was revealed that the high-TMB group exhibited a poorer prognosis compared to the low-TMB group. Further stratification of patients according to both the risk score and TMB yielded intriguing findings in **Figure 8B**. Specifically, it was observed that the low-TMB and high-EAS group experienced the most unfavorable prognosis. In the cohorts receiving immunotherapy, namely GSE91061, GSE100797, GSE126044, and GSE35640, a comparative analysis demonstrated that the majority of patients in the low-EAS group exhibited a significantly higher proportion of treatment responders when compared to the high-EAS group. The statistical significance of these differences was assessed using various methods, including the Bonferroni adjusted value, the FDR adjusted value, and the Nominal p value, with the majority of the disparities found to be statistically significant (**Figure 8C**).

### 3.8 Enrichment analysis and immunization checkpoints

**Figure 9:**
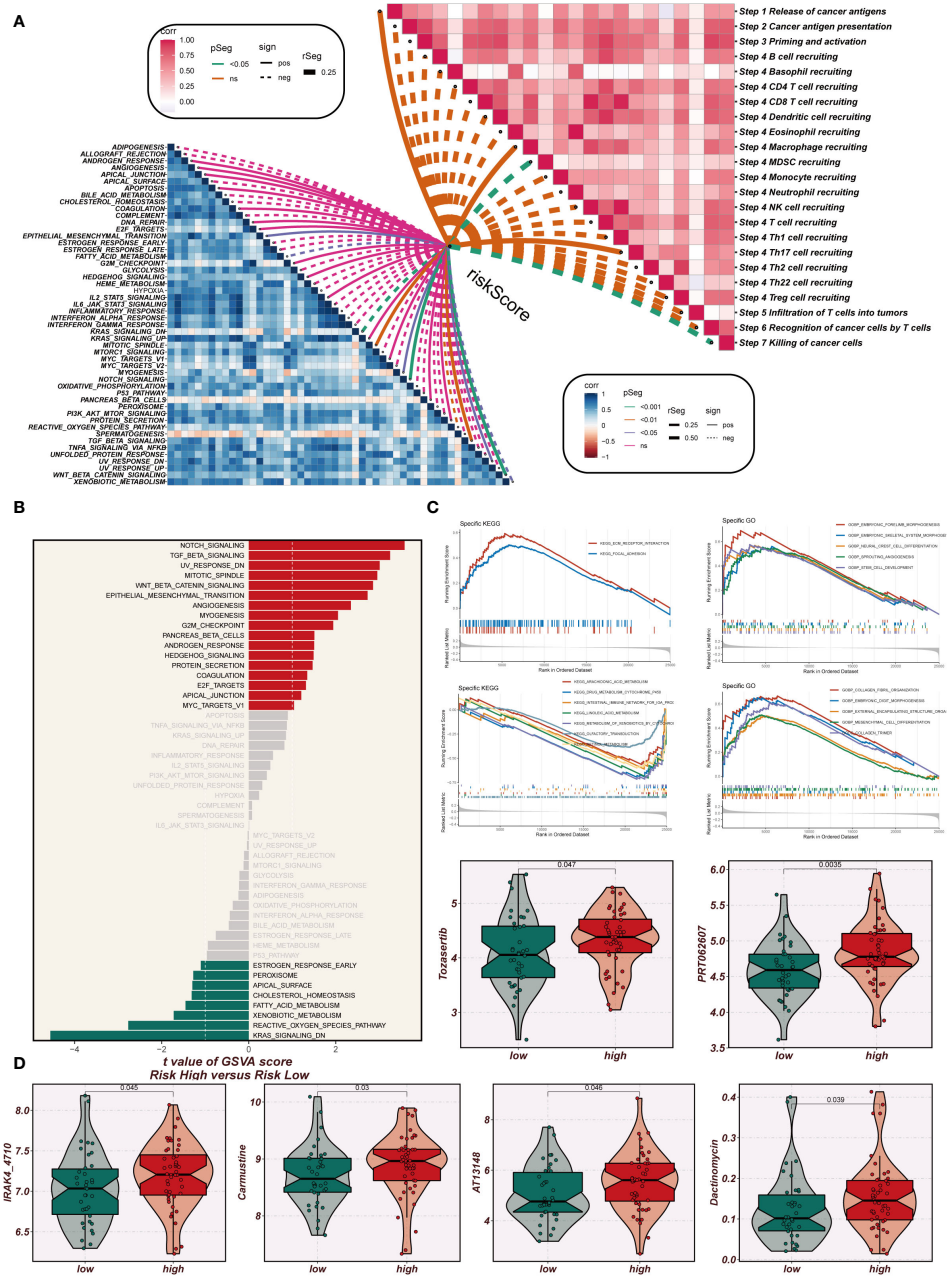
Enrichment analysis and immunotherapy analysis. **(A)** The relationship between risk scores and the steps of tumor immune cycle and hallmark gene sets. **(B)** GSVA enrichment analysis demonstrates the enrichment of hallmark gene sets between high- and low-risk groups. **(C)** GSEA enrichment analysis showed the enrichment of different genes in the GO and KEGG pathways between different risk groups. **(D)** Box plots comparing the sensitivity of high- and low-risk groups to six chemotherapeutic agents.

A comprehensive correlation analysis was undertaken between the risk score and hallmark gene sets, as well as the cancer immunity cycle, revealing a clear negative association between the risk score and most components of the cancer immunity cycle. Notably, in the hallmark-related analysis, a positive correlation was observed between the risk score and specific pro-oncogenic pathways, including DNA repair, E2F targets, and G2M checkpoint (**Figure 9A**). Signaling pathway differences in different risk groups were assessed using marker gene sets. **Figure 9B** illustrates that enrichment in signaling pathways such as Notch signaling, TGF beta signaling, angiogenesis, and G2M checkpoint was primarily observed in the high-EAS group. Conversely, the low-EAS group demonstrated enrichment in KRAS signaling, the reactive oxygen species pathway, and fatty acid metabolism. To further explore these findings, GO and KEGG enrichment analyses were conducted using the GSEA method. KEGG enrichment analysis indicated significant enrichment in pathways associated with ECM receptor interaction and focal adhesion for the high-EAS group. In contrast, enrichment in pathways related to arachidonic acid metabolism, linoleic acid metabolism, KRAS signaling, the reactive oxygen species pathway, and fatty acid metabolism was observed in the low-EAS group. Additionally, GO enrichment analysis highlighted significant enrichment in pathways related to embryonic forelimb morphogenesis, embryonic skeletal system morphogenesis, sprouting angiogenesis, and collagen for the high-EAS group. Notably, substantial enrichment was observed in sprouting angiogenesis and collagen fibril organization pathways (**Figure 9C**). Potential effective chemotherapeutic agents for different risk groups were explored using the “oncopredict” R package. The results identified six drugs, namely Tozasertib, PRT062607, IRAK4_4710, Carmustine, AT13148, and Dactinomycin, which may hold greater efficacy as potential antitumor agents for low-EAS patients (**Figure 9D**).

### 3.9 Vitro experimental validation

**Figure 10:**
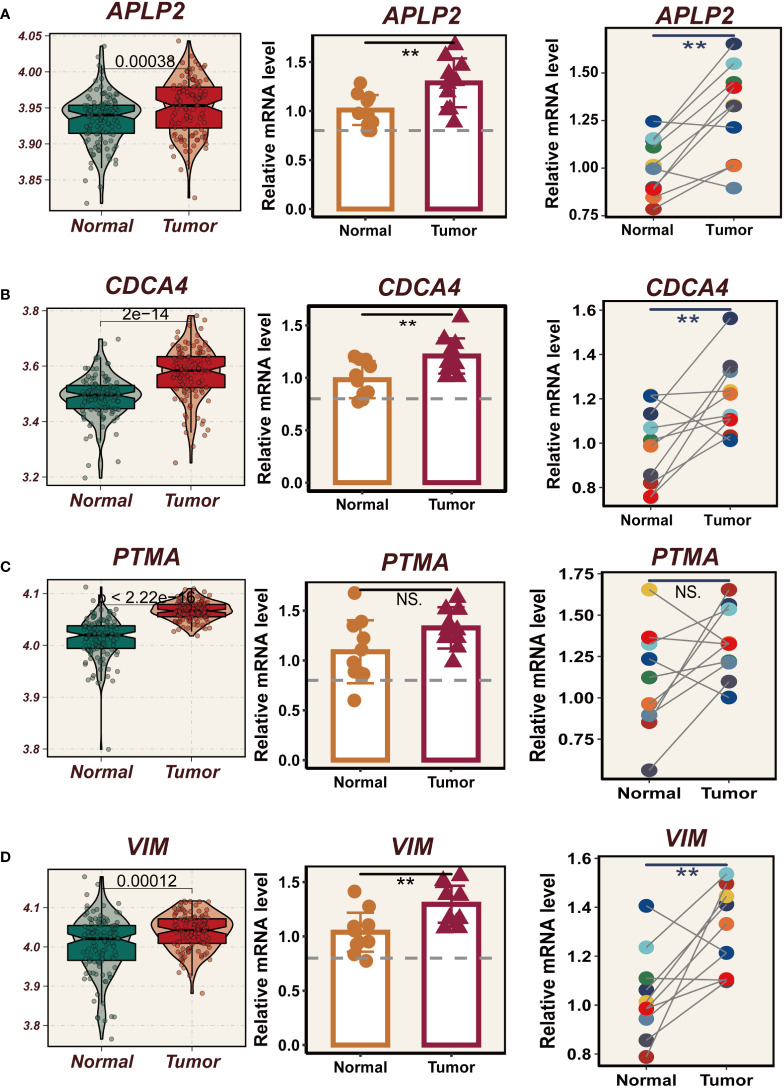
Experimental validation of model gene. **(A)** Box plots showing differential expression of APLP2 in tumor and normal tissues in TCGA-ESCC;10 relative expression of APLP2 gene in pairs of cancer and paracancer samples. **(B)** Box plots showing differential expression of CDCA4 in tumor and normal tissues in TCGA-ESCC; 10 relative expression of CDCA4 gene in pairs of cancer and paracancer samples. **(C)** Box plots showing differential expression of PTMA in tumor and normal tissues in TCGA-ESCC;10 relative expression of PTMA gene in pairs of cancer and paracancer samples. **(D)** Box plots showing differential expression of VIM in tumor and normal tissues in TCGA-ESCC;10 relative expression of VIM gene in pairs of cancer and paracancer samples. **P < 0.01; ns, P > 0.05.

**Figure 11:**
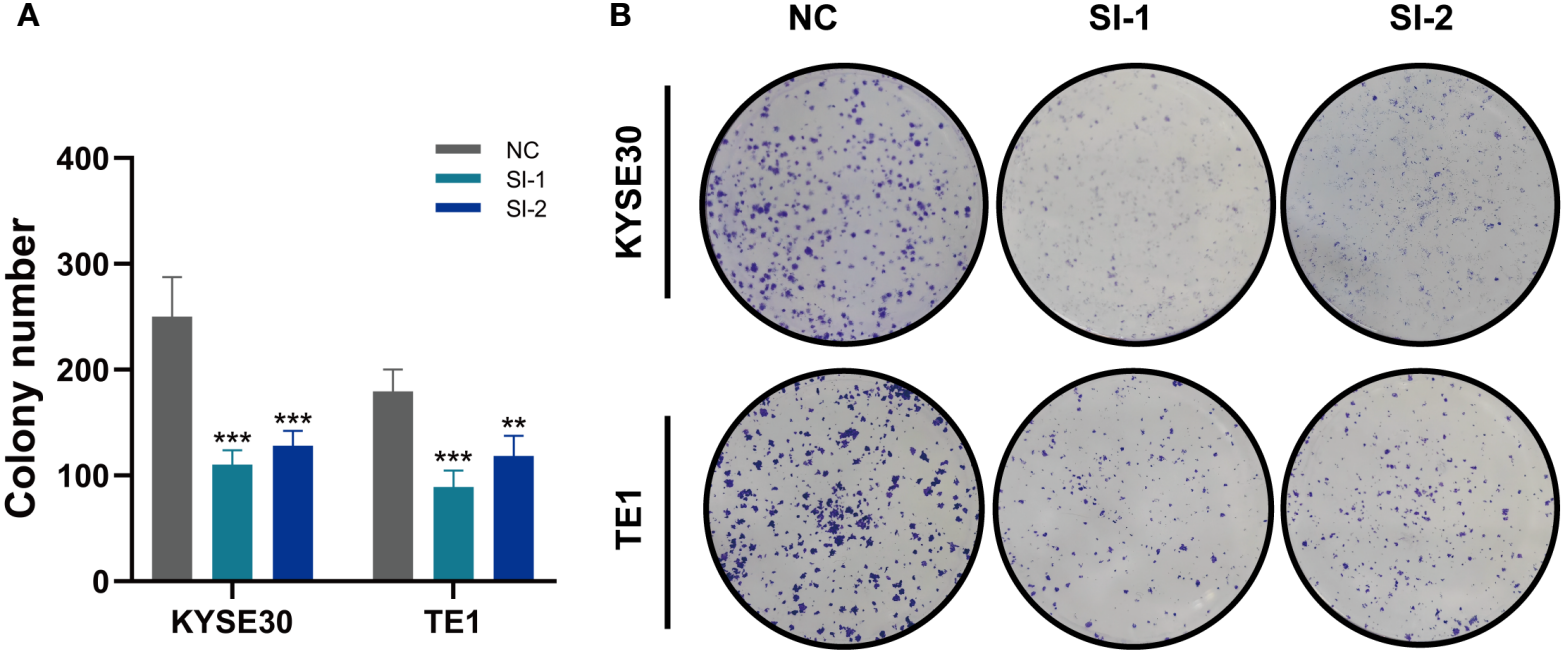
*In vitro* Experiment **(A, B)** CCK-8 detection and colony formation assays show that inhibition of APLP2 expression significantly suppressed the proliferation of ESCC cells. **P < 0.01; ***P < 0.001.

In TCGA, the expression levels of APLP2, CDCA4, PTMA and VIM were significantly different between normal and tumor samples with HR>1, while the other model genes showed no significant difference or small HR (**Supplementary Figure 2B**). To further validate these four model genes, qRT-PCR was performed using surgically resected tumor tissues and normal esophageal tissues, and it was found that the expression of APLP2, CDCA4, and VIM genes was significantly up-regulated in the tumor tissues, whereas the expression of the PTMA gene was also up-regulated but not statistically different (**Figures 10A-D**). Furthermore, we used siRNA to inhibit the expression of APLP2 in KYSE30 and TE1 cells. CCK-8 and colony formation assays revealed that the inhibition of APLP2 significantly suppressed the proliferation capacity of ESCC cells (**Figures 11A, B**). **Supplementary Figure 2A** showed that immune checkpoint genes such as CD44, HHLA2, PDCD1 and TNFRSF18 were significantly different in both high- and low-EAS group, with CD44 showing high expression in the high-EAS group, whereas HHLA2, PDCD1 and TNFRSF18 were more highly expressed in the low-EAS group, suggesting that the effect of immunotherapy in the low-EAS group may be better.

## 4 Discussion

Esophageal cancer (EC), ranking 8th in incidence and 6th in mortality globally, poses a severe risk. With the current incidence rates, an estimated 957,000 new cases of EC are projected by 2040 (1, 27, 28). Unfortunately, many patients are diagnosed at advanced stages, leading to dismal 5-year survival rates (2). Immunotherapy has emerged as a promising option for various cancers, including EC (29–31). This innovative approach leverages the immune system to combat malignant cells, inhibiting tumor progression. However, individual responses vary, and complications may arise. Precise molecular characterization is urgently needed for targeted anti-tumor therapies (3).

In this study, all esophageal cancer squamous epithelial cells were classified into three clusters using the UMAP dimensionality reduction algorithm, and then 20 model genes related to ESCC prognosis were obtained by COX regression and Lasso regression analysis of cluster mark genes, and EAS were constructed based on them. Based on the EAS, patients were divided into high- and low-EAS group, and the survival analysis found that the prognosis of the high-EAS group was significantly worse. ROC curve analysis was performed on the training and test groups and found that the AUC values of the TCGA cohort and the GEO53624 validation cohort were above 0.7, showing good discriminatory ability. The model was applied to four immunotherapy cohorts (GSE91061, GSE100797, GSE126044, GSE35640) and found that patients in the low-EAS group had better immunotherapy outcomes. The results of drug sensitivity tests showed that Tozasertib, PRT062607, IRAK4_4710, Carmustine, AT13148 and Dactinomycin could be potential agents for esophageal cancer treatment. In addition, we performed qRT-PCR *in vitro* validation and found that APLP2,CDCA4 and VIM genes were significantly overexpressed in tumor tissues, and the expression of PTMA gene was also upregul XCated, but the difference lacked statistical significance.

APLP2, located on chromosome 16, is a gene that encodes the APLP2 protein. The APLP2 protein is a type I transmembrane protein involved in crucial cellular processes such as migration, adhesion, proliferation, and signaling. Previous research has highlighted the dysregulation of APLP2 in various cancer types, including colorectal, lung, breast, and pancreatic cancers (32–35). Its involvement in abnormal growth, invasion, and metastasis has been observed. However, there is inconsistency regarding the expression pattern (increase or decrease) of APLP2 in different tumors, and the precise underlying reasons and resulting effects remain unknown (36). Notably, a study by Tao et al. focused on hepatocellular liver cancer and constructed a predictive model based on four disulfide apoptotic differential genes, including APLP2. This model demonstrated high predictive performance in multiple cohorts, revealing that APLP2 influences the oncogenic processes of hepatocellular liver cancer by regulating apoptosis and the cell cycle (37). Gao et al. investigated renal cell carcinoma and found that APLP2 expression serves as an independent predictor of survival prognosis (P=0.026), indicating its significance in patient survival and prognosis (38). Additionally, Poelaert et al. identified increased APLP2 expression in pancreatic cancer epithelium compared to pancreatic intraepithelial neoplasia epithelial cells. This finding was further validated in a KPC mouse model, suggesting that APLP2 could be a potential therapeutic target for pancreatic cancer (39). In the present study, the expression of APLP2 in esophageal cancer tissues was found to be significantly higher than in normal tissues, and this observation was confirmed by qPCR analysis.

CDCA4 is a gene that encodes a protein with crucial functions in regulating the cell cycle, controlling E2F-dependent transcriptional activation, and governing cell proliferation. Its role in cell division is of significant importance (40). Previous studies conducted using cellular and animal models have demonstrated the association of CDCA4 with various malignant tumors. In breast cancer, non-small cell lung cancer, osteosarcoma, and squamous cell carcinoma of the head and neck, CDCA4 has been found to be up-regulated (41–44). In the realm of nephroblastoma, Li et al. discovered that CDCA4 exhibited high expression levels and played a role in promoting cell proliferation while inhibiting apoptosis. This effect was mediated through the activation of the AKT/mTOR signaling pathway (45). Furthermore, Zheng et al. constructed a prognostic map for esophageal cancer, utilizing eight genes, including CDCA4, UBE2Z, AMTN, AK1, TLE1, FXN, ZBTB6, and APLN. This columnar map holds promise in providing valuable insights for precise clinical management of the disease (46).

The PTMA gene encodes a small acidic protein that is widely distributed throughout the body and possesses notable pro-tumorigenic characteristics. This protein exerts inhibitory effects on apoptosis while promoting tumor cell proliferation. High expression of PTMA has been associated with a poorer prognosis in several tumor types, including esophageal, bladder, melanoma, hepatocellular, and gallbladder cancers (47–51). In addition to its intracellular functions, PTMA can also be secreted extracellularly and act as a damage-associated molecular pattern (DAMP) during cellular stress and infection. Under such circumstances, PTMA exhibits diverse immunomodulatory functions, including its role in anti-tumor immunity (52). Shao et al. conducted a study utilizing weighted gene co-expression network analysis (WGCNA) to identify differentially expressed genes and key modules contributing to the development and progression of ESCC. Their findings suggest that the PTMA gene may serve as a potential prognostic marker for ESCC (53). Another investigation by Chen et al. found that PTMA expression is significantly elevated in ESCC compared to normal tissues. Inhibition of PTMA expression was shown to substantially reduce the activity of ESCC cells while promoting apoptosis. Furthermore, PTMA was found to bind to HMGB1, influencing mitochondrial oxidative phosphorylation and impacting the malignant progression of ESCC (54). In our present study, we observed overexpression of the PTMA gene in esophageal cancer tissues, which was further validated through *in vitro* experiments. These findings underscore the potential of PTMA as a target for immunotherapy in the treatment of EC.

The VIM gene encodes an intermediate filament protein that belongs to the family of cytoskeletal proteins. This protein plays a crucial role in providing structural support and regulating various cellular functions. Overexpression of VIM has been consistently associated with key features of tumor progression, including invasion, metastasis, and resistance of tumor cells. Consequently, elevated VIM expression is considered one of the hallmarks of tumor development and prognosis (55, 56). In the context of gliomas, Liu et al. made an intriguing discovery linking high expression of VIM with negative patient survival outcomes. They also observed a positive correlation between VIM expression and immune infiltration as well as tumor progression. These findings suggest that VIM could potentially serve as a target for immunotherapy in the treatment of gliomas (57). In a study by Lien et al. focused on invasive low-stage endometrial carcinoma, they found that lower expression of epithelial waveform protein and VIM gene correlated with poorer recurrence-free survival. The loss or low expression of VIM was identified as a potent FIGO stage I recurrence marker, emphasizing its prognostic significance in this particular cancer type (58). In summary, the aforementioned four genes play vital roles in the development of ESCC and warrant further investigation.

Two specific subgroups that markedly influence the prognosis of ESCC patients have been identified through an investigation into the heterogeneity within malignant epithelial cell subgroups of esophageal cancer. A prognostic prediction model for ESCC has been constructed using 20 distinctive genes within these subgroups, showcasing a high degree of stability and accuracy, as validated in an external dataset. This model is positioned as a robust tool for the clinical treatment of ESCC, offering personalized treatment options tailored to individual circumstances of patients.

## Data availability statement

The datasets presented in this study can be found in online repositories. The names of the repository/repositories and accession number(s) can be found in the article/**Supplementary Material**.

## Ethics statement

The studies involving humans were approved by The Ethics Committee of The Affiliated Huaian No.1 People’s Hospital. The studies were conducted in accordance with the local legislation and institutional requirements. The participants provided their written informed consent to participate in this study.

## Author contributions

CL: Conceptualization, Methodology, Writing – original draft. WS: Conceptualization, Software, Supervision, Writing – review & editing. JZ: Data curation, Formal Analysis, Validation, Writing – review & editing. YL: Conceptualization, Formal Analysis, Supervision, Writing – review & editing.

## Supplementary material

Supplementary Figure 1 The expression of four regulons in each cluster were showed in Violin plot and UMAP plot.

Supplementary Figure 2
**(A)** Box plots showing differential expression of 4 immunization checkpoint genes in tumor and normal tissues in TCGA-ESCC. **(B)** Box plots showing differential expression of 16 model genes in tumor and normal tissues in TCGA-ESCC.
