# Supplementary figures and images for: Single-cell transcriptomics reveals heterogeneity in esophageal squamous epithelial cells and constructs models for predicting patient prognosis and immunotherapy
Source: Front Immunol. 2023 Nov 30;14:1322147. doi: 10.3389/fimmu.2023.1322147 (PMC10719955; doi:10.3389/fimmu.2023.1322147)

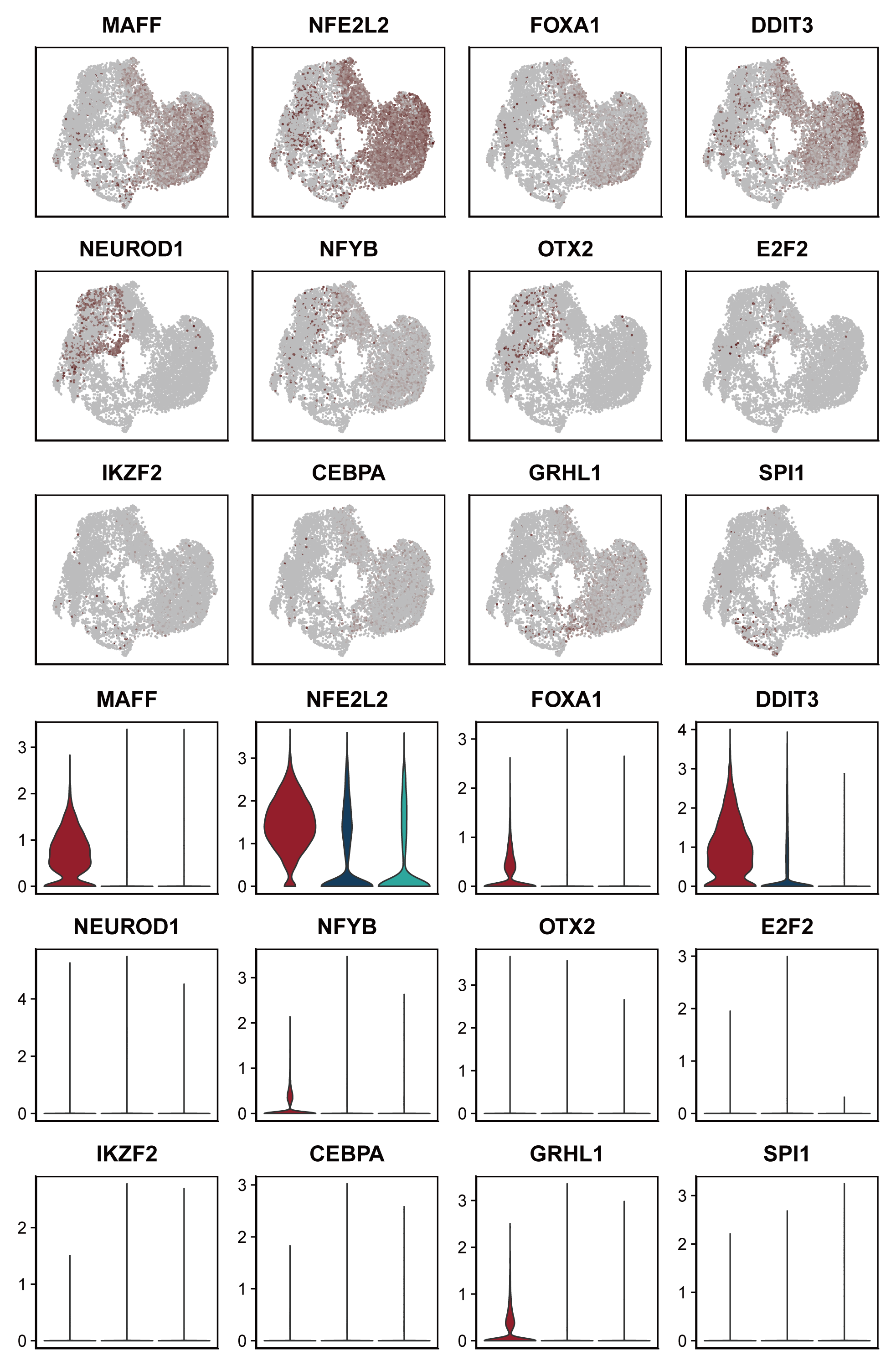

Supplement: Supplementary Figure 1 — The expression of four regulons in each cluster were showed in Violin plot and UMAP plot. [file Image_1.tif]

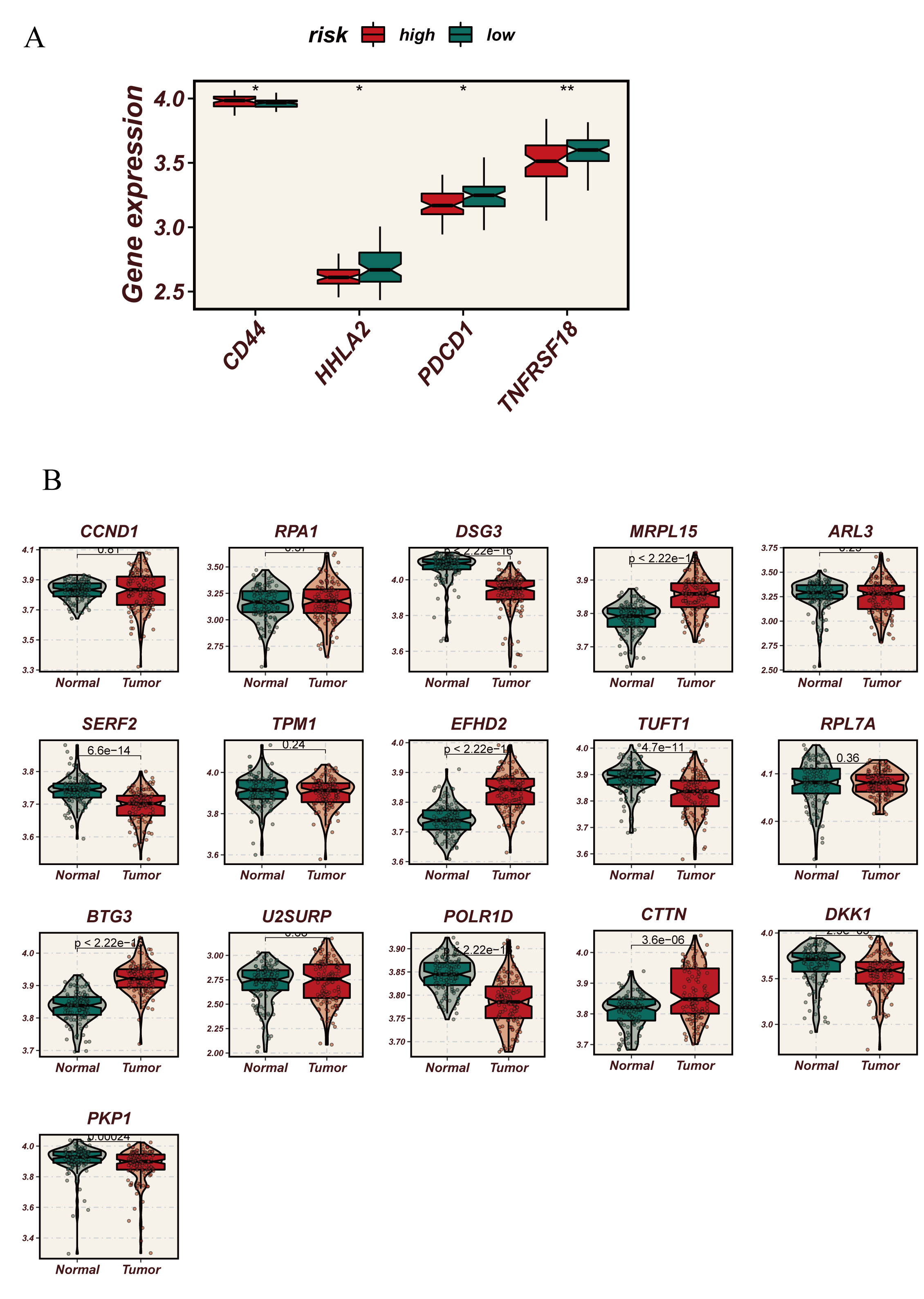

Supplement: Supplementary Figure 2 — (A) Box plots showing differential expression of 4 immunization checkpoint genes in tumor and normal tissues in TCGA-ESCC. (B) Box plots showing differential expression of 16 model genes in tumor and normal tissues in TCGA-ESCC. [file Image_2.tif]
